# The Effects of Transcranial Electrical Stimulation of the Brain on Sleep: A Systematic Review

**DOI:** 10.3389/fpsyt.2021.646569

**Published:** 2021-06-07

**Authors:** Clément Dondé, Jerome Brunelin, Jean-Arthur Micoulaud-Franchi, Julia Maruani, Michel Lejoyeux, Mircea Polosan, Pierre A. Geoffroy

**Affiliations:** ^1^University Grenoble Alpes, Grenoble, France; ^2^U1216 INSERM, Grenoble Institut of Neuroscience, La Tronche, France; ^3^Psychiatry Department, CHU Grenoble Alpes, Grenoble, France; ^4^INSERM U1028, CNRS UMR5292, Lyon Neuroscience Research Center, PSY-R2 Team, Lyon, France; ^5^Lyon University, Lyon, France; ^6^Centre Hospitalier le Vinatier, Batiment 416, Bron, France; ^7^University Sleep Clinic, Services of Functional Exploration of the Nervous System, University Hospital of Bordeaux, Bordeaux, France; ^8^USR CNRS 3413 SANPSY, University Hospital Pellegrin, University of Bordeaux, Bordeaux, France; ^9^Département de Psychiatrie et de Médecine Addictologique, Hôpital Fernand Widal, Assistance Publique des Hôpitaux de Paris (APHP), Paris, France; ^10^Université de Paris, Paris, France; ^11^INSERM U1144, Optimisation Thérapeutique en Neuropsychopharmacologie, Paris, France; ^12^Paris Diderot University-Paris VII, 5 Rue Thomas Mann, Paris, France; ^13^University Hospital Bichat-Claude Bernard, 46 rue Henri Huchard, Paris, France; ^14^Université de Paris, NeuroDiderot, Inserm, Paris, France

**Keywords:** sleep, transcranial electrical stimulation, sleep oscillations, sleep pattern, subjective sleep, systematic review

## Abstract

Transcranial Electrical Stimulation (tES) is a promising non-invasive brain modulation tool. Over the past years, there have been several attempts to modulate sleep with tES-based approaches in both the healthy and pathological brains. However, data about the impact on measurable aspects of sleep remain scattered between studies, which prevent us from drawing firm conclusions. We conducted a systematic review of studies that explored the impact of tES on neurophysiological sleep oscillations, sleep patterns measured objectively with polysomnography, and subjective psychometric assessments of sleep in both healthy and clinical samples. We searched four main electronic databases to identify studies until February 2020. Forty studies were selected including 511 healthy participants and 452 patients. tES can modify endogenous brain oscillations during sleep. Results concerning changes in sleep patterns are conflicting, whereas subjective assessments show clear improvements after tES. Possible stimulation-induced mechanisms within specific cortico-subcortical sleep structures and networks are discussed. Although these findings cannot be directly transferred to the clinical practice and sleep-enhancing devices development for healthy populations, they might help to pave the way for future researches in these areas. PROSPERO registration number 178910.

## Introduction

Sleep plays a vital role in well-being and good health throughout life. It is essential for many brain processes including the consolidation of memories (1, 2), alertness, processing speed, and decision-making (3). Healthy regulation of these processes by sleep has a significant relationship with scores of quality of life and global functioning (4). Sleep disorders, as medically defined, have significant public health implications, with insomnia complaints reported by nearly one third of the general population, and excessive daytime sleepiness affecting up to one fourth (5). In addition, it has been demonstrated that alteration of sleep is a marker of risk for numerous physical and mental disorders (6, 7). Abnormal fluctuations in sleep duration and efficiency (8–10), regularity of sleep cycle (11), subjective sleep quality (12), attentive wakefulness (13), and timing of sleep (14) are associated with greater risk of adverse general health outcomes including death. More specifically, sleep disruptions are associated with higher risk of diabetes, stroke, coronary heart disease and heart attack (10, 15), obesity (16), as well as mental disorders (7). Moreover, sleep complaints are often integral and important parts of a large range of diagnosed chronic medical conditions (17, 18).

Several tools have been developed to measure and assess objective and subjective aspects of sleep. Electroencephalography (EEG) studies have provided numerous indicators of the sleep course and quality. For instance, slow-wave oscillations have been demonstrated as accurate indexes of sleep homeostasis (19). In parallel, sleep patterns including architecture, timing, and stages differentiation can be measured with standardized polysomnography. Finally, several psychometric rating scales and brief interviews have been validated to assess subjective aspects of sleep and are widely used in clinical trials as main outcomes (20).

The functioning of brain areas and networks can be modified by applying electrical currents over the scalp. To modulate brain activity and eventually restore altered functions, transcranial Electrical Stimulation (tES) has recently emerged as a non-invasive painless brain modulation tool that involves the application of a weak (0.5–2 mA) current via two scalp electrodes (a cathode and an anode) overlying targeted cortical areas (21, 22). The electrical current flowing between the electrodes induces changes in neuronal excitability and activity through specific molecular mechanisms that mediate synaptic plasticity (23). tES studies have traditionally used direct current modalities for stimulation (tDCS: transcranial direct stimulation), where a constant unidirectional low current flows inward under the anode and outward under the cathode. Besides, other stimulation modalities have been developed for tES involving random noise frequencies (tRNS) or alternating (tACS) patterns of the current (24, 25). In healthy samples, an increasing number of studies have reported that tES can enhance various cognitive functions including memory, learning and attention, especially during learning of the task (26–28). In parallel, tDCS has shown promising results in treating psychiatric disorders such as major depressive disorder (29), various clinical symptoms and cognitive impairments in bipolar disorder (30, 31), auditory hallucinations (32), and negative symptoms (33) in schizophrenia, as well as numerous others (34).

Arousal and sleep are physiologically modulated by “top-down” cortico-subcortical loops (35) that are known to be altered in some sleep disorders such as insomnia (36). The “top-down” concept raises the idea of modulating sleep with external stimulation of the neocortex. Given that tES has primarily cortical direct effects, this approach appears thus as a relevant therapeutic strategy. Over the past years, there have been several attempts to modulate sleep with tES-based approaches targeting “top-down” networks in both the healthy and pathological brains. However, in addition to heterogeneous and inconsistent results, studies strongly differ in terms of the number of samples, tES protocols, and type of measures. Furthermore, despite a growing interest in sleep modulations with electric current, data about the impact of tES in sleep remain scattered between studies and have not been systematically reviewed, which prevent from drawing definitive conclusions on the effect of tES on measurable aspects of sleep.

To gather knowledge about the specific effects of brain tES on core measurable aspects of sleep, we conducted a systematic review of studies that explored the impact of such procedure on neurophysiological sleep oscillations, sleep patterns measured objectively with polysomnography and subjective psychometric assessments of sleep. Here, we focus on the specific impact of tES on these sleep-related outcomes in both healthy and clinical populations; for details about the effect of tES during sleep on wake cognitive processes, we refer the interested reader to the exhaustive review recently published by Barham et al. (37). Results presented in our review will contribute to the understanding of underlying mechanisms of tES on sleep and improvement of sleep health, sleep complaints, and sleep disorders with tES. In parallel, this review may lend insight for further enhancement of sleep induction/stability, as well as physiological effects of sleep (e.g., memory strengthening) with innovative techniques of neuromodulation in healthy people.

## Methods

### Eligibility

Recommendations of the PRISMA guidelines for systematic review and meta-analysis were followed (38). The protocol was prospectively registered at the PROSPERO register (ID: 178910) (39).

The criteria for inclusion were as follows: (i) English language studies published in peer-review journals, (ii) participants' age from 10–80 years. (iii) oscillatory data measured with electroencephalography and/or sleep pattern data reported from polysomnographic (or actigraphy) recordings and/or subjective sleep data assessed with validated rating scale, and (iv) measures collected pre-to-post and/or during the tES procedure. If two publications reported findings from the same dataset, the authors were contacted to identify the most appropriate data to review.

### Literature Search Strategy

We searched the MEDLINE, Embase, ScienceDirect, and Clinical Trials databases using the following Medical Subject Heading (MeSH) terms in reference title, abstract, or keywords with no limitation of date until February 2020:

- (i) *Sleep*: “sleep,” “insomnia,” “hypersomnia,” “sleepiness,” “sleep apnea,” “somnolence,” “snoring,” “restless legs syndrome,” “periodic limb movements disorder,” “REM sleep behavior disorder,” “obstructive sleep apnea,” “sleep-wake disruption,” “parasomnia,” “bruxism,” “circadian rhythm sleep disorder.”
- (ii) *tES*: “Transcranial stimulation,” “tES,” “Transcranial Electrical Stimulation,” “tDCS,” “Transcranial Direct Current Stimulation,” “tRNS,” “Transcranial Random Noise Stimulation,” “tACS,” “Transcranial Alternating Current Stimulation.”

**Figure 1:**
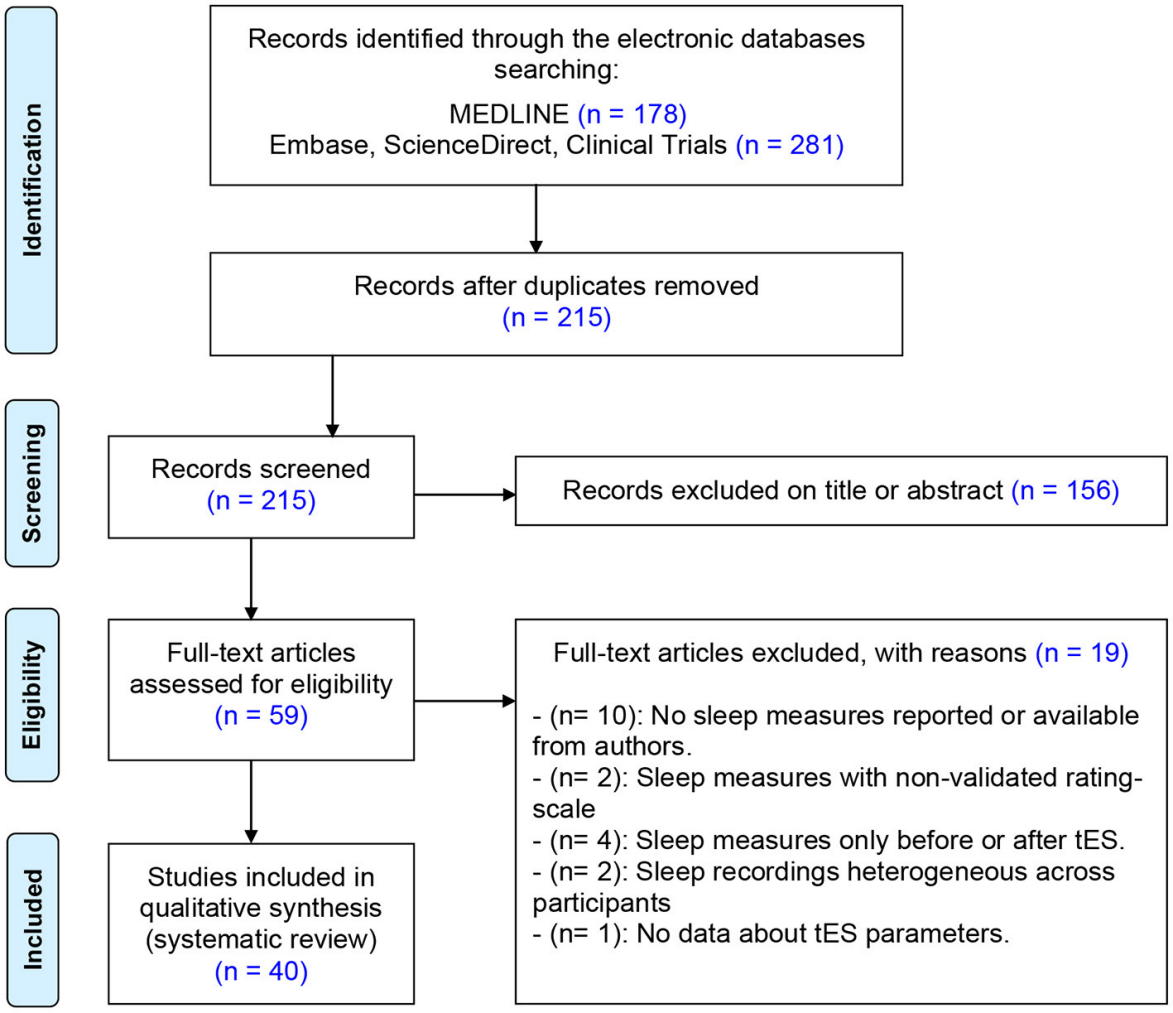
Flow diagram for the systematic review.

After excluding duplicate references, two reviewers (CD and PAG) independently screened the title and abstract of each study identified by the search and applied the inclusion criteria. Following this first screen, we applied the same procedure to the full text of eligible studies. Discrepancies between reviewers were resolved by discussion with a third member of the authorship. The “similar articles” findings in MEDLINE and Reference lists in identified studies were also reviewed for additional studies, although none were identified in this manner. The literature search strategy is detailed in the flow chart diagram (Figure 1).

### Data Extraction

Two reviewers (CD and PAG) independently extracted the following data when present: (i) population data (sample size, gender ratio, disorder, or healthy status), (ii) study design (parallel or crossover groups, controlled or uncontrolled), (iii) tES protocol (anode and cathode placement; electrode size; current density and intensity; number, frequency, and duration of sessions; period and condition of stimulation), (iv) neurophysiological outcomes as measured with electroencephalography and/or sleep pattern outcomes as measured with polysomnographic (or actigraphy) recordings and/or subjective sleep outcomes as assessed with validated rating scale, and (v) adverse effects of the tES procedure.

### Quality Assessment

To measure the overall quality of the included references, a global rating score was calculated for each study using the Standard Quality Assessment [QualSyst tool (40)].

## Results

### Literature Search

**Figure 2:**
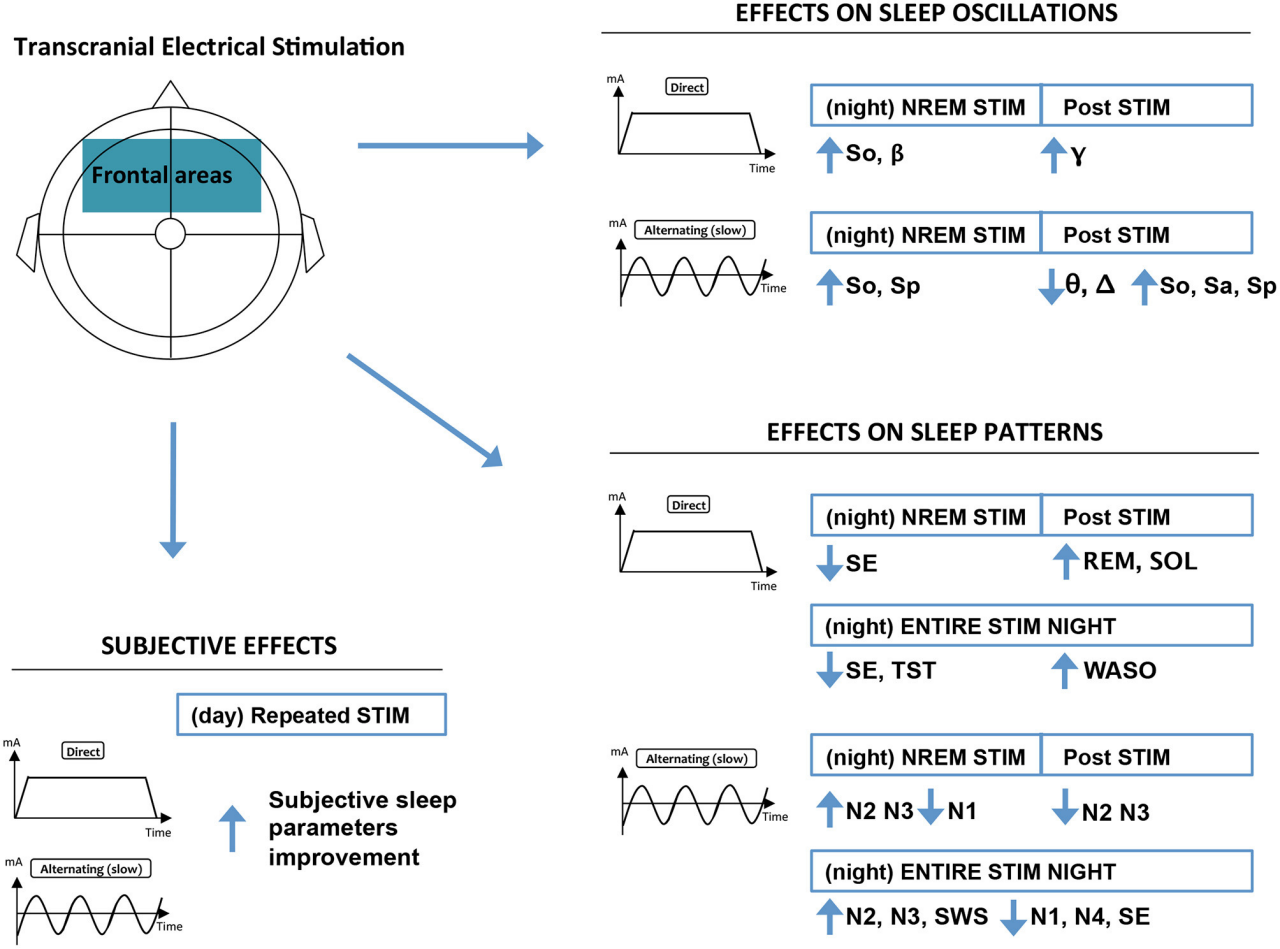
Highlights of major significant effects of frontal tES on sleep. Results regarding non-frontal tES, tES during REM, and fast alternating tES were scarce, and thus omitted from the figure. Effect on sleep patterns: NREM, non-rapid eye movement sleep; N1–4, NREM sleep stages relative duration; REM, rapid eye movement sleep; SE, sleep efficiency; SWS, slow wave sleep relative duration; SOL, sleep onset latency; WASO, wake after sleep onset. Effects on sleep oscillations: β, beta power (15–25 Hz); Δ, delta power (1–4 Hz); γ, gamma power (>25 Hz); θ, theta power (4–8 Hz); NREM, non-rapid eyes movement sleep; So, slow oscillations power (0.5–1 Hz); Sa, slow activity power (0.5–4 Hz); Sp, spindle power (10–15 Hz).

As shown in Figure 1, the initial search returned 459 references after duplicate removal. Following preliminary screening of the titles and/or abstracts, 215 were excluded accordingly. Among the 59 references that were reviewed in detail, 40 studies were selected for systematic review including 511 healthy participants and 452 patients. The age of the participants varied from 12.3 to 73.4 years. Only 8 studies out of 40 reported transient adverse effects associated to the tES procedure (41–48). Overall, the quality assessment was satisfactory (mean: 22.1 ± 3.99). Total scores and details of the assessment are given in the Supplementary Table 1. Highlights of major significant effects of tES on sleep are pictured in Figure 2 and detailed in Supplementary Table 2.

### Oscillatory Aspects of Sleep

#### Study Designs and Characteristics

Twenty-four studies investigated the impact of tES on oscillatory aspects of sleep in healthy populations (45, 47, 49–64) and clinical populations (65–70). All used a crossover sham-controlled design with days-to-week washout periods between conditions, with the exception of two studies that included parallel arms of participants (60, 69). Study details are described in Table 1.

**Table 1:** Impact of transcranial electrical stimulation on oscillatory aspects of sleep.

**References**	**Design**	**tES montage**	**tES sessions**	**Outcomes**	**Significant findings**	**Side effects**
**Placebo-controlled parallel-arms**						
Garside et al. (53)	8 healthy (21.0 ± 0.93) 1 active arm, 1 sham arm	*Electrodes*: 5 cm^2^ *Anodes*: 2 anterior–posterior to the left DLPFC (C3), 2 contralateral *Cathode*: 2 anterior–posterior to the left mastoid, 2 contralateral *Intensity*: 0.55 mA *Current*: alternating (0.75 Hz)	*Number*: 1 *Duration*: 30 min (5-min cycles with 1-min free intervals) *Period*: daytime nap N2 or N3	*Waves:* slow oscillations (0.7–0.8 Hz), delta (1–4 Hz), theta (4–8 Hz), alpha (9–12 Hz), fast spindle (12–14 Hz) *Parameters:* PSD	*Stimulation-free intervals:* decreased (reduced normal increase) delta power at Fz, C3, C4 by active vs. sham *1-min post-stimulation interval:* no significant changes	NA
Roizenblatt et al. (69)	*Frontal-active arm:* 11 fibromyalgia (47.3 ± 11.0 age, 11F) *Motor-active arm:* 11 fibromyalgia (54.8 ± 9.3 age, 11F) *Sham arm*: 10 fibromyalgia (50.8 ± 10.2 age, 10F)	*Electrodes*: 25 cm^2^ *Active frontal arm: Anode*: right primary motor area (C3), *Cathode:* left supraorbital area (Fp2) *Active motor arm: Anode*: left DLPFC (C3), *Cathode:* right supraorbital area (Fp1) *Intensity*: 2 mA *Type*: direct	*Number*: 5 *Frequency:* daily *Duration*: 20 min *Period*: daytime before night sleep	*Waves:* delta (1–4 Hz), alpha (8–12 Hz) *Parameters:* PSD	*N1–N4 night sleep after stimulation procedure:* increased alpha and decreased delta at all electrodes by frontal-active vs. sham; increased delta by motor-active vs. sham.	None reported
Antonenko et al. (49)	15 healthy (23.4 ± 1.9 age, 7F) 1 active arm, 1 sham arm 4-week washout	*Electrodes*: 0.8 cm^2^ *Anodes*: 2, DLPFC (F3, F4) *Cathodes:* 2 extra-cerebral (bilateral mastoids) *Intensity*: 0.32 mA/cm^2^ *Current*: alternating (0.75 Hz squared)	*Number*: 1 *Duration*: 30 min (4-min cycles with 1-min free intervals) *Period*: 4 min after night N2 or N3 onset	*Waves:* slow activity (0.5–4 Hz), slow spindle (9–12 Hz), fast spindle (12–15 Hz), beta (15–25 Hz) *Parameters:* PSD, PAC	*Stimulation-free intervals:* no significant results *Stimulation intervals*: coupling of slow activity up-phases with spindle waves at Pz by active vs. sham.	NA
Cellini et al. (50)	17 healthy (32.2 ± 8.1 age, 6F) 1 active arm, 1 sham arm 1-week washout	*Electrodes*: 40 cm^2^ *Anodes*: 2 anterior–posterior to the left DLPFC (C3), 2 contralateral *Cathode*: 2 anterior–posterior to the left mastoid, 2 contralateral *Intensity*: 2 mA *Current*: alternating (0.75 Hz)	*Number*: 1 *Duration*: 4-s cycles 5 s after endogenous slow-oscillations *Period*: daytime nap N2 or N3	*Waves:* slow oscillations (0.75 Hz), fast spindle (12–15 Hz), slow spindle (9–12 Hz) *Parameters:* PSD	*Stimulation-free intervals:* increased slow oscillations at Fz, Fp1, FP2, F7, FC1 by active vs. sham.	NA
Del Felice et al. (65)	12 with temporal lobe epilepsia (34.2 ± 15.4 age, 8F) 1 active arm, 1 sham arm 1-week washout	*Electrodes*: 0.8 cm^2^ *Anodes*: affected temporal lobe (F7-T3 or F8-T8) *Cathode*: ipsilateral mastoid *Intensity*: 0.25 mA *Current*: alternating (0.75 Hz)	*Number*: 1 *Duration*: 30 min (5-min cycles with 1-min free intervals) *Period*: daytime before nap	*Waves:* slow spindle (10–12 Hz), fast spindle (12–14 Hz) *Parameters:* PSD	*Entire post-stimulation sleep period:* increased slow spindle power at all electrodes by active vs. sham.	NA
Eggert et al. (51)	23 healthy (69.3 ± 8.0 years; 14F) 1 active arm, 1 sham arm 1-week washout	*Electrodes*: NA *Anodes*: 2, DLPFC (F3, F4) *Cathodes:* 2 extra-cerebral (bilateral mastoids) *Intensity*: 0.33 mA/cm^2^ *Current*: alternating (0.75 Hz)	*Number*: 1 *Duration*: five 5/16-min cycles with 1-min free intervals *Period*: after night N3 onset	*Waves*: slow oscillation (0.5–1 Hz), delta (1–4 Hz), theta (4–8 Hz), alpha (8–11 Hz), slow spindle (11–13 Hz), fast spindle (13–15 Hz), beta (15–25 Hz) *Parameters:* PSD	*Stimulation-free intervals:* decreased delta power at F7 and T3 electrodes by active vs. sham *60-min post-stimulation interval*: no significant results *Entire night*: no significant results	NA
Frase et al. (52)	19 healthy (53.7 ± 6.9 age, 13F) 1 anodal-active arm; 1 cathodal-active arm; 1 sham arm 1-week washout	*Electrodes*: 35 cm^2^ (anode), 100 cm^2^ (cathode) *Anodal-active arm:* *Anode*: 2 supraorbital areas (Fp1, Fp2) *Cathode*: 2 parietal (P3, P4) *Cathodal-active arm:* *Cathode*: 2 supraorbital areas (Fp1, Fp2) *Anode*: 2 parietal (P3, P4) *Intensity*: 2 mA *Current*: direct	*Number*: 2 with 20-min inter-stimulation interval *Duration*: 13 min (anodal), 9 min (cathodal) *Period*: daytime before night sleep	*Waves*: delta (0.1–3.5 Hz), theta (3.5–8 Hz), alpha (8–12 Hz), sigma (12–16 Hz), beta (16–24 Hz), gamma (24–50 Hz) *Parameters:* PSD	*NREM night sleep*: increased beta power at C3-A2 electrodes by active cathodal (vs. anodal and sham) during first quarter of the night. *5-min interval after stimulation night*: increased gamma power at C3-A2 electrodes by anodal (vs. cathodal), decrease gamma power at C3-A2 electrodes after cathodal (vs. sham and anodal) *5-min interval 24 h after stimulation night*: increased gamma power C3-A2 electrodes by anodal (vs. cathodal).	NA
Frase et al. (66)	19 insomnia disorder (43.8 ± 15.1 age, 13F) 1 anodal-active arm; 1 cathodal-active arm; 1 sham arm 1-week washout				*N1-N4 night sleep*: decreased delta power at Fz electrodes by anodal vs. sham and cathodal vs. sham. *5-min interval after stimulation night*: no significant changes *5-min interval 24 h after stimulation night*: no significant changes	NA
Johnson and Durrant (45)	15 healthy (20.7 ± 0.3 age, 10F) 1 5 Hz active arm; 1 0.75 Hz active arm; 1 sham arm 2-day washout	*Electrodes*: 2.5 cm^2^ (cathode), 24 cm^2^ (anode) *Cathode*: right DLPFC F4 *Anode*: extra-cranial *Intensity*: 0.4-mA *Current:* alternating (5 Hz or 0.75 Hz)	*Number*: 1 *Duration*: 25 min *Period*: 4 min after night REM sleep onset	*Waves*: slow activity (0.5–2 Hz), theta (4–8 Hz) *Parameters:* PSD	*Slow wave and REM night sleep:* no significant changes.	NA
Ketz et al. (54)	16 healthy (22.3 ± 5.0 age, 3F) 1 active arm, 1 sham arm 1-week washout	*Electrodes*: NA *Anodes*: 2, DLPFC (F3, F4) *Cathodes:* 2 extra-cerebral (bilateral mastoids) *Intensity*: 1.5 mA *Current*: alternating (0.5–1.2 Hz)	*Number*: 1 *Duration*: periods of 5 slow-wave cycles throughout the night *Period*: 4 min after N2 sleep onset, during slow oscillations	*Waves:* slow oscillations (0.5–1.2 Hz) *Parameters:* PSD, PAC	*10-s post-stimulation interval:* increased (+3–4 s) then decreased (+4–10 s) slow oscillation power, and increased coupling with spindle amplitude of slow oscillations at all electrodes by active vs. sham	NA
Koo et al. (55)	25 healthy (22.4 ± 2.1 15F) 1 active arm, 1 sham arm, 1-week washout	*Electrodes*: 0.5 cm^2^ *Anodes*: 2, DLPFC (F3, F4) *Cathodes:* 2 extra-cerebral (bilateral mastoids) *Intensity*: NA *Type*: alternating 0.84 Hz	*Number*: 1 *Duration*: 30 min (5-min cycles with 1-min free intervals) *Period*: during N2	*Waves:* slow oscillation (0.5–1.5 Hz), delta (1.5–4 Hz), theta (4–8 Hz), slow spindle (9–12 Hz), fast spindle (12–15 Hz). *Parameters:* PSD, length	*Stimulation-free intervals:* no significant differences *150-min post-stimulation interval*: increased fast spindle PSD and length at C and P electrodes by active vs. sham	NA
Ladenbauer et al. (67)	16 mild cognitive impairment (70.6 ± 8.9 age, 7F) 1 active arm, 1 sham arm, 2-week washout	*Electrodes*: 0.5 cm^2^ *Anodes*: 2, DLPFC (F3, F4) *Cathodes:* 2 extra-cerebral (bilateral mastoids) *Intensity*: 0.52 mA/cm^2^ *Type*: alternating 84 Hz	*Number*: 1 *Duration*: 30 min (5-min cycles with 100-s free intervals) *Period*: during N2	*Waves:* slow oscillation (0.5–1.5 Hz), slow spindle (8–12 Hz), fast spindle (12–15 Hz). *Parameters:* PSD	*Stimulation-free intervals:* increased slow oscillation and fast spindle at C and P electrode by active vs. sham. *Stimulation intervals*: coupling of slow oscillation up-phases with fast spindle at F, C, and P electrodes by active vs. sham.	NA
Lustenberger et al. (47)	15 healthy males 1 active arm, 1 sham arm	*Electrodes*: 35 cm^2^ *Anode*: 1 left DLPFC (F3) *Cathode*: 1 right DLPFC (F4) *Intensity*: 0.16 mA/cm^2^ *Current:* alternating 12 Hz	*Number*: 1 *Duration*: cycles of a few seconds only when spindle activity (11–16 Hz) is prevailing *Period*: night N2/N3	*Waves*: delta (1–4 Hz), theta (4–8 Hz), fast spindle (11–16 Hz) *Parameters:* PSD	*Stimulation-free intervals:* increased spindle power, decreased delta and theta power at all electrodes by active vs. sham	1 in the active group (first-degree burn)
Marshall et al. (58)	18 healthy (19–28 age) 1 active arm, 1 sham arm 1-week washout	*Electrodes*: 0.5 cm^2^ *Anodes*: 2 anterior–posterior to the left DLPFC (C3), 2 contralateral *Cathode*: 2 anterior–posterior to the left mastoid, 2 contralateral *Intensity*: 0.26 mA/cm^2^ *Current*: direct	*Number*: 1 *Duration*: 30 min (sixty 15-s stimulation alternating with sixty 15-s free intervals) *Period*: 30 s after night N3 or N4 onset	*Waves:* slow oscillation (0.5–1 Hz), delta (1–4 Hz), theta (4–8 Hz), alpha (8–12 Hz), fast spindle (12–15 Hz), beta (20–25 Hz) *Parameters:* PSD	*Stimulation-free intervals:* increased slow-oscillations at P electrode and delta at F and P electrodes by active vs. sham *N2:* no significant changes *N3-N4*: decreased theta, alpha, and beta at C, F, and P by active vs. sham	NA
Marshall et al. (56)	13 healthy 1 active arm, 1 sham arm 10 week washout	*Electrodes*: 0.5 cm^2^ *Anodes*: 2 anterior–posterior to the left DLPFC (C3), 2 contralateral *Cathode*: 2 anterior–posterior to the left mastoid, 2 contralateral *Intensity*: 0.52 mA/cm^2^ *Current*: alternating (0.75 Hz)	*Number*: 1 *Duration*: 30 min (5-min cycles with 1-min free intervals) *Period*: 4 min after night N2 onset	*Waves:* slow oscillation (0.5–1 Hz), delta (1–4 Hz), slow spindle (8–12 Hz), fast spindle (12–15 Hz) *Parameters:* PSD	*Stimulation-free intervals:* increased slow oscillations (power) and slow spindle PSD at Fz by active vs. sham	NA
Marshall et al. (57)	25 healthy (18–35 age) 1 N2-active arm, 1 REM-active arm, 1 sham arm 10-day washout	*Electrodes*: 0.5 cm^2^ *Anodes*: 2 anterior–posterior to the left DLPFC (C3), 2 contralateral *Cathode*: 2 anterior–posterior to the left mastoid, 2 contralateral *Intensity*: 0.52 mA/cm^2^ *Current*: alternating (5 Hz)	*Number*: 1 *Duration*: 30 min (5-min cycles with 1-min free intervals) *Period*: 4 min after night N2 or REM onset	*Waves:* slow oscillation (0.5–1 Hz), delta (1–4 Hz), theta (4–8 Hz), slow spindle (8–12 Hz), fast spindle (12–15 Hz), beta (12–25 Hz), gamma (25–45 Hz) *Parameters:* PSD	*Stimulation-free intervals:* decreased slow oscillations and delta power at C, F, and P electrodes, and decreased slow spindles power at Fz by N2-active vs. sham; increased gamma power at C, F, and P during REM-active vs. sham *First 30-min post-stimulation interval:* increased slow oscillations power at C, F, and P and increased slow spindles power at Fz electrode during by N2 active vs. sham; *Second 30-min post-stimulation interval*: no significant results	NA
Munz et al. (68)	14 ADHD (12.3 ± 1.4 age) 1 active arm, 1 sham arm, 1-week washout	*Electrodes*: 0.5 cm^2^ *Anodes*: 2, DLPFC (F3, F4) *Cathodes:* 2 extra-cerebral (bilateral mastoids) *Intensity*: 0.52 mA/cm^2^ *Type*: alternating 0.75 Hz	*Number*: 1 *Duration*: 30 min (5-min cycles with 1-min free intervals) *Period*: 4 min after N2 onset	*Waves*: slow oscillations *Parameters:* PSD	*Stimulation-free intervals:* increased slow oscillations power at all electrodes by active vs. sham. *60-min post-stimulation interval*: no significant results	NA
Passman et al. (59)	21 healthy (65.0 ± 1.0 age, 10F) 1 active arm, 1 sham arm, 2/3-week washout	*Electrodes*: 0.5 cm^2^ *Anodes*: 2, DLPFC (F3, F4) *Cathodes:* 2 extra-cerebral (bilateral mastoids) *Intensity*: 0.52 mA/cm^2^ *Type*: alternating 0.75 Hz	*Number*: 1 *Duration*: 30 min (5-min cycles with 1-min free intervals) *Period*: 4 min after N2 onset	*Waves*: slow oscillation (0.5–1 Hz), slow spindle (8–12 Hz), fast spindle (12–15 Hz) *Parameters:* PSD	*Stimulation-free intervals:* increased slow oscillations and slow spindle power at F electrodes; increased fast spindle power at C and P electrodes by active vs. sham. *1 min after 60-min post-stimulation interval*: increased slow oscillations power at F electrodes,	NA
Reato et al. (60)	*Active arm*: 13 healthy, *Sham arm*: 10 healthy	*Electrodes*: NA *Anodes*: 2, DLPFC (F3, F4) *Cathodes:* 2 extra-cerebral (bilateral mastoids) *Intensity*: 0.26 mA *Current*: alternating (0.75 Hz)	*Number*: 1 *Duration*: 30 min (5-min cycles with 1-min free intervals) *Period*: night N2 or N3	*Waves:* slow oscillations (0.5–1 Hz), slow activity (0.5–4 Hz) *Parameters:* PSD, spatial coherence	*Entire post-stimulation sleep period*: increased (reduced normal decrease) slow oscillations and slow activity (PSD and spatial coherence) at all electrodes by active vs. sham	NA
Saebipour et al. (70)	6 insomnia (34 ± 7 age, 2F) 1 active arm, 1 sham arm, 1-day washout	*Electrodes*: 0.5 cm^2^ *Anodes*: 2, DLPFC (F3, F4) *Cathodes:* 2 extra-cerebral (bilateral mastoids) *Intensity*: NA *Type*: alternating 0.75 Hz	*Number*: 1 *Duration*: 30 min (5-min cycles with 1-min free intervals) *Period*: 4 min after N2 onset	*Waves*: slow oscillation (0.1–1 Hz) *Parameters:* PSD	*Stimulation-free intervals:* increased slow oscillation at C3 by active vs. sham Post-stimulation interval: increased probability of transition from N2 to N3, decreased probability of transition from N3 to wakefulness	NA
Sahlem et al. (61)	12 healthy (25.0 age, 9F) 1 active arm, 1 sham arm 1-day washout	*Electrodes*: 1.13 cm^2^ *Anodes*: 2, DLPFC (F3, F4) *Cathodes:* 2 extra-cerebral (bilateral mastoids) *Intensity*: 0.6 mA *Current*: alternating (0.75 Hz squared)	*Number*: 1 *Duration*: 30 min (5-min cycles with 1-min free intervals) *Period:* 4 min after night N2 or N3 onset	*Waves*: slow oscillation (0.5–1 Hz), delta (1–4 Hz), theta (4–8 Hz) slow spindle (8–12 Hz), fast spindle (12–15 Hz), beta (15–25 Hz) *Parameters:* PSD	*Stimulation-free intervals:* no significant results *60-min post-stimulation interval*: no significant results	NA
Venugopal et al. (62)	12 healthy (29.8 ± 6.2 age, 12M), 1 active-N2 arm, 1 active-REM arm 1-day washout	*Electrodes*: 4 cm^2^ *Anodes*: 2, DLPFC (F3, F4) *Cathodes:* 2 extra-cerebral (bilateral mastoids) *Intensity*: 0.2 mA *Current*: alternating (0.75 Hz for active-N2, 40 Hz for active-REM protocol)	*Number*: 1 *Duration*: 15 min (four 30-s cycles with 3-min free intervals) *Period*: 1 min after night N2 or REM onset	EEG power before/after stimulation *Waves:* slow activity (0.5–4 Hz), theta (4–8 Hz), alpha (8–12 Hz), beta (12–30 Hz) *Parameters:* PSD	*30-s post-stimulation interval*: no significant changes	NA
Voss et al. (63)	27 healthy (18–26 age, 15F) 7 active and 7 corresponding sham conditions counter-balanced across 4 consecutive nights	*Electrodes*: 14 cm^2^ *Anodes*: 2, DLPFC (F3, F4) *Cathodes:* 2 extra-cerebral (bilateral mastoids) *Intensity*: 0.25 mA *Current*: alternating (2, 6, 12, 25, 40, 70, or 100 Hz)	*Number*: 1 *Duration*: 30 s *Period*: 2 min after REM sleep onset	*Waves:* from 0 to 100 Hz *Parameters:* PSD	*Stimulation intervals:* no significant changes	NA
Westerberg et al. (64)	19 healthy (73.4 age, 16F) 1 active arm, 1 sham arm 1-week washout	*Electrodes*: 0.50-cm^2^ *Anodes*: 2, DLPFC (F7, F8) *Cathodes:* 2 extra-cerebral (bilateral mastoids) *Intensity*: NA *Current*: alternating (0.75 Hz)	*Number*: 1 *Duration*: 30 min (5-min cycles with 1-min free intervals) *Period:* 4 min after daytime nap N2 or N3 onset	*Waves*: slow oscillation (0.5–1 Hz), delta (1–4.5 Hz), slow spindle (8.5–13.5 Hz), fast spindle (13.5–15.5 Hz) *Parameters:* PSD	*Stimulation-free intervals:* increased slow oscillations PSD at F electrodes; decreased fast spindle PSD at F electrodes by active vs. sham *60-min post-stimulation interval*: no significant results	NA

*DLPFC, dorsolateral prefrontal cortex; NA, not available; NREM 1–4, Non-Rapid Eye Movement Sleep (four stages of non-rapid eye movement sleep, each progressively going into deeper sleep); REM, Rapid Eye Movement Sleep (unique stage of sleep characterized by random rapid movement of the eyes, low muscle tone throughout the body and propensity of the sleeper to dream vividly)*.

*Sleep oscillations parameters: PAC (PAC), measure of the coupling between the phase of low-frequency rhythms and the amplitude of higher-frequency oscillations; Power Spectral Density (PSD), measure of frequency content (energy per unit time) vs. frequency; Length, reflects the full extension (ms) of the time-frequency bin on the time axis; Spatial coherence, correlation (or predictable relationship) between waves at different points in space*.

#### Results From Studies in Healthy Populations

Most research groups aimed for “top-down” effects and gave a single tES session targeting bilateral frontal areas with returning electrodes overlying the mastoids or the vertex. In these studies, the stimulation applied cycles of slow alternating current (around 0.75 Hz, interspersed by stimulation-free intervals) that were triggered during early stages NREM sleep (47, 49, 51, 55–57, 59, 61, 64, 67, 68). This specific design was employed after Marshall et al. original study to test if the prefrontal slow oscillations enhancement by tES during NREM is associated with increased hippocampal–neocortical-dependent declarative memory consolidation. This hypothesis is based on evidence that slow oscillations are the hallmark of neurophysiological activity during NREM being associated with neutral declarative memory (58). A subsequent enhancement of slow oscillations PSD by direct (58) and slow alternating (50, 56, 59, 64) tES vs. sham was repeatedly observed during stimulation-free intervals at the stimulated areas. In addition, increased spindle waves PSD (56, 59) and decreased delta/theta PSD (51, 53) were reported during these intervals. Another study from Marshall's group that involved higher-frequency alternating current (5 Hz) observed opposite patterns of changes, i.e., decreased slow oscillations, delta, and slow spindle power by tES (57). In contrast, one experiment using a slow spindle-like alternating current (12 Hz) observed an increase of power after tES in this specific frequency range (47).

An increase in slow oscillations and spindle waves power by active tES against sham was mainly demonstrated by several studies during long intervals after both slow-wave (0.75 Hz) and theta (5 Hz) alternating NREM stimulation (46, 57, 59, 60). However, recordings during post-stimulation periods were highly heterogeneous in terms of intervals where oscillations were measured and averaged. Regarding per-stimulation recordings, a single study using specific filtering procedures demonstrated a significant coupling between NREM slow oscillations up-phases and spindle waves at parietal electrodes concomitant to active tES vs. sham (49). No significant changes in sleep oscillations were observed when taking into consideration the entire stimulation night (51). In parallel, some research groups stimulated the brain with slow alternating current during REM (rapid-eye movement sleep). An increase in gamma power during stimulation-free intervals was demonstrated (57), but no significant changes were reported during stimulation intervals and entire sleep periods (45, 63).

Two studies used a bifrontal tES montage involving direct stimulating current (52, 58). It was observed that direct cathodal stimulation during daytime before sleep increased beta power at central sites during the first NREM night sleep cycle vs. both anodal and sham stimulation. By contrast, anodal stimulation induced an increase in gamma band power after the stimulation night compared with the other stimulation conditions (52). In parallel, Marshall and colleagues demonstrated that direct tES concomitant to NREM increases delta power in stimulation-free intervals and decreases higher-frequency band power during post-stimulation periods of slow wave sleep (58). No long-term effects were investigated across studies.

#### Results From Studies in Clinical Populations

Six studies investigated the effect of a single brain tES session on oscillatory aspects of sleep in clinical populations. All were sham-controlled crossover trials. Two studies involved patients with insomnia disorder. Decreased NREM delta power after a single frontal anodal direct stimulation during daytime (66) and increased slow oscillation power during NREM stimulation-free intervals by alternating slow current vs. sham were observed in this population (70). In parallel, an increase of slow oscillation power during NREM stimulation-free intervals by alternating slow current vs. sham was reported in a group of patients with ADHD (Attention Deficit/Hyperactivity Disorder) (68). A similar tES montage also increased slow oscillations in patients with mild cognitive impairment, along with increased spindle power during NREM stimulation-free intervals (67). A three-arm study involving patients with fibromyalgia reported an increased alpha and decreased delta power after five sessions of frontal stimulation vs. sham and an increased delta power after five sessions of motor stimulation vs. sham (69). Finally, stimulation of the affected temporal lobe of patients with temporal lobe epilepsy before a nap induced an increase of slow spindle wave power (65).

### Sleep Patterns

#### Study Designs and Characteristics

Twenty studies investigated the impact of tES on sleep patterns in healthy (45, 47, 49–52, 55–61, 63, 64) and clinical (65–70) populations. Studies explored tES-induced changes in patterns of sleep including continuity (e.g., total sleep time, sleep efficiency, wake after sleep onset, and sleep onset latency) and architecture (i.e., sleep stages relative duration—expressed as percentage of total sleep time—and latency). Study details are described in Table 2.

**Table 2:** Impact of transcranial electrical stimulation on pattern aspects of sleep.

**References**	**Sample and design**	**tES montage**	**tES sessions**	**Outcomes**	**Significant findings**	**Adverse effects**
**Placebo-controlled parallel-arms**						
Roizenblatt et al. (69)	*Active frontal arm:* 11 fibromyalgia (47.3 ± 11.0 age, 11F) *Active motor arm:* 11 fibromyalgia (54.8 ± 9.3 age, 11F) *Sham arm*: 10 fibromyalgia (50.8 ± 10.2 age, 10F)	*Electrodes*: 25 cm^2^ *Active frontal arm: Anode*: right primary motor area (C3), *Cathode:* left supraorbital area (Fp2) *Active motor arm: Anode*: left DLPFC (C3), *Cathode:* right supraorbital area (Fp1) *Intensity*: 2 mA *Type*: direct	*Number*: 5 *Frequency:* daily *Duration*: 20 min *Period*: 1 week	*Sleep continuity:* AI, SE, SOL, and TST *Sleep architecture:* N1, N2, N3, N4, REM relative duration	*Entire night after stimulation procedure:* decreased SE, increased SOL and increased REM relative duration by frontal-active vs. sham; increased SE, decreases arousals by motor-active vs. sham.	None reported
Lustenberger et al. (47)	16 healthy males 1 active arm, 1 sham arm	*Electrodes*: 35 cm^2^ *Anode*: 1 left DLPFC (F3) *Cathode*: 1 right DLPFC (F4) *Intensity*: 4 mA *Current:* alternating 12 Hz	*Number*: 1 *Period*: during night N2 (spindle activity 11–16 Hz)	*Sleep continuity:* SE, SOL, TST, WASO *Sleep architecture:* N1, N2, N3, REM relative duration	*Entire night:* no significant changes.	NA
**Placebo-controlled crossover**						
Cellini et al. (50)	17 healthy (32.2 ± 8.1 age, 6F) 1 active arm, 1 sham arm 1-week washout	*Electrodes*: 0.8 cm^2^ *Anodes*: 2 anterior–posterior to the left DLPFC (C3), 2 contralateral *Cathode*: 2 anterior–posterior to the left mastoid, 2 contralateral *Intensity*: 2 mA *Current*: alternating (0.75 Hz)	*Number*: 1 *Duration*: 4-s cycles 5 s after endogenous slow oscillations *Period*: night N2 or N3	*Sleep continuity:* SE, SOL, TST, WASO *Sleep architecture:* N1, N2, SWS (N3), REM relative duration	*Entire stimulation night*: increased N3 relative duration and decreased N1 relative duration by active vs. sham	NA
Del Felice et al. (65)	12 with temporal lobe epilepsia (34.2 ± 15.4 age, 8F) 1 active arm, 1 sham arm 1-week washout	*Electrodes*: 0.8 cm^2^ *Anodes*: affected temporal lobe (F7-T3 or F8-T8) *Cathode*: ipsilateral mastoid *Intensity*: 0.25 mA *Current*: alternating (0.75 Hz)	*Number*: 1 *Duration*: 30 min (5-min cycles with 1-min free intervals) *Period*: daytime before nap	*Sleep continuity:* SOL, TST *Sleep architecture:* N1, N2, N3, REM relative duration	*Entire post-stimulation nap:* increased TST and decreased SOL by active vs. sham.	NA
Voss et al. (63)	27 healthy (18–26 age, 15F) 7 active and 7 corresponding sham conditions counter-balanced across 4 consecutive nights	*Electrodes*: 14 cm^2^ *Anodes*: 2, DLPFC (F3, F4) *Cathodes:* 2 extra-cerebral (bilateral mastoids) *Intensity*: 0.25 mA *Current*: alternating (2, 6, 12, 25, 40, 70, or 100 Hz)	*Number*: 1 *Duration*: 30 s *Period*: 2 min after REM sleep onset	*Sleep continuity:* SE, SOL, TST, WASO *Sleep architecture:* N1, N2, SWS (N3), REM relative duration	*Entire stimulation night*: no significant changes.	NA
Marshall et al. (58)	18 healthy (19–28 age) 1 active arm, 1 sham arm 1-week washout	*Electrodes*: 0.5 cm^2^ *Anodes*: 2 anterior–posterior to the left DLPFC (C3), 2 contralateral *Cathode*: 2 anterior–posterior to the left mastoid, 2 contralateral *Intensity*: 0.26 mA/cm^2^ *Current*: direct	*Number*: 1 *Duration*: 30 min (sixty 15-s stimulation alternating with sixty 15-s free intervals) *Period*: 30-s after night N3 or N4 onset	*Sleep continuity:* TST, WASO *Sleep architecture:* N1, N2, SWS (N3+N4), REM relative duration; N2 and SWS latency	*Entire stimulation night*: no significant changes.	NA
Marshall et al. (56)	13 healthy 1 active arm, 1 sham arm 1-week washout	*Electrodes*: 0.5 cm^2^ *Anodes*: 2 anterior–posterior to the left DLPFC (C3), 2 contralateral *Cathode*: 2 anterior–posterior to the left mastoid, 2 contralateral *Intensity*: 0.52 mA/cm^2^ *Current*: alternating (0.75 Hz)	*Number*: 1 *Duration*: 30 min (5-min cycles with 1-min free intervals) *Period*: 4-min after night N2 onset	*Sleep continuity:* WASO *Sleep architecture:* N1, N2, SWS (N3+N4) relative duration	*Entire stimulation night*: increased SWS relative duration by 0.75-Hz active vs. sham.	NA
Marshall et al. (57)	25 healthy (18–35 age) 1 N2-active arm, 1 REM-active arm, 1 sham arm 10-day washout	*Electrodes*: 0.5 cm^2^ *Anodes*: 2 anterior–posterior to the left DLPFC (C3), 2 contralateral *Cathode*: 2 anterior–posterior to the left mastoid, 2 contralateral *Intensity*: 0.52 mA/cm^2^ *Current*: alternating (5 Hz)	*Number*: 1 *Duration*: 30 min (5-min cycles with 1-min free intervals) *Period*: 4 min after night N2 or REM onset	*Sleep continuity:* SOL, TST, WASO *Sleep architecture:* N1, N2, SWS (N3+N4), REM relative duration; SWS and REM latency	*Entire stimulation night*: increased SWS latency by N2-active vs. sham *Stimulation free intervals*: increased N2 relative duration, decreased SWS relative duration by N2-active vs. sham *30-min post-stimulation*: decreased N2 relative duration by N2-active vs. sham	NA
Antonenko et al. (49)	15 healthy (23.4 ± 1.9 age, 7F) 1 active arm, 1 sham arm 4-week washout	*Electrodes*: 0.8 cm^2^ *Anodes*: 2, DLPFC (F3, F4) *Cathodes:* 2 extra-cerebral (bilateral mastoids) *Intensity*: 0.32 mA/cm^2^ *Current*: alternating (0.75 Hz squared)	*Number*: 1 *Duration*: 30 min (4-min cycles with 1-min free intervals) *Period*: 4 min after night N2 or N3 onset	*Sleep continuity:* AI, SE, SOL, TST, WASO *Sleep architecture:* N1, N2, N3, N4, REM relative duration	*Entire stimulation night*: no significant changes	NA
Westerberg et al. (64)	19 healthy (73.4 age, 16F) 1 active arm, 1 sham arm 1-week washout	*Electrodes*: 0.50 cm^2^ *Anodes*: 2, DLPFC (F7, F8) *Cathodes:* 2 extra-cerebral (bilateral mastoids) *Intensity*: NA *Current*: alternating (0.75 Hz)	*Number*: 1 *Duration*: 30 min (5-min cycles with 1-min free intervals) *Period:* 4 min after nap N2 or N3 onset	*Sleep continuity:* SE, SOL, WASO *Sleep architecture:* N1, N2, REM, SWS (N3+N4) relative duration	*Entire stimulation night*: no significant changes	NA
Eggert et al. (51)	23 healthy (69.3 ± 8.0 years; 14F) 1 active arm, 1 sham arm 1-week washout	*Electrodes*: NA *Anodes*: 2, DLPFC (F3, F4) *Cathodes:* 2 extra-cerebral (bilateral mastoids) *Intensity*: 0.33 mA/cm^2^ *Current*: alternating (0.75 Hz)	*Number*: 1 *Duration*: five 5/16-min cycles with 1-min free intervals *Period*: early night N3	*Sleep continuity:* WASO *Sleep architecture:* N1, N2, SWS (N3+N4), REM relative duration	*Entire stimulation night*: no significant changes *Stimulation-free intervals*: increased WASO relative duration and decreased N3 relative duration by active vs. sham *60-min post-stimulation*: no significant changes	NA
Frase et al. (52)	19 healthy (53.7 ± 6.9 age, 13F) 1 anodal-active arm; 1 cathodal-active arm; 1 sham arm 1-week washout	*Electrodes*: 35 cm^2^ (anode), 100 cm^2^ (cathode) *Anodal-active arm: Anode*: 2 supraorbital areas (Fp1, Fp2)*, Cathode*: 2 parietal (P3, P4) *Cathodal-active arm: Cathode*: 2 supraorbital areas (Fp1, Fp2)*, Anode*: 2 parietal (P3, P4) *Intensity*: 2 mA *Current*: direct	*Number*: 2 with 20-min inter-stimulation interval *Duration*: 10 min *Period*: day before night sleep	*Sleep continuity:* AI, SE, SOL, TST, WASO *Sleep architecture:* N2, SWS (N3), REM relative duration; REM latency, REM cycles,	*Entire stimulation night*: decreased SE and TST; increased WASO relative duration by anodal-active (vs. cathodal-active and sham); increased REM relative duration by cathodal-active (vs. sham)	NA
Johnson and Durrnt (45)	15 healthy (20.7 ± 0.3 age, 10F) 1.5 Hz active arm; 1 0.75 Hz active arm; 1 sham arm 2-day washout	*Electrodes*: 2.5 cm^2^ (cathode), 24 cm^2^ (anode) *Cathode*: right DLPFC F4 *Anode*: extra-cranial *Intensity*: 0.4 mA *Current:* alternating (5 Hz or 0.75 Hz)	*Number*: 1 *Duration*: 25 min *Period*: 4 min after night REM sleep onset	*Sleep continuity:* SE, TST *Sleep architecture:* N1, N2, SWS, REM relative duration	*Entire stimulation night:* decreased SE by 0.75 Hz active (vs. 5 Hz active and sham)	None reported
Sahlem et al. (61)	12 healthy (25.0 age, 9F) 1 active arm, 1 sham arm 1-week washout	*Electrodes*: 1.13 cm^2^ *Anodes*: 2, DLPFC (F3, F4) *Cathodes:* 2 extra-cerebral (bilateral mastoids) *Intensity*: 0.6 mA *Current*: alternating (0.75 Hz squared)	*Number*: 1 *Duration*: 30 min (5-min cycles with 1-min free intervals) *Period:* 4 min after N2 or N3 onset	*Sleep continuity:* SE, TST *Sleep architecture:* N1, N2, SWS (N3+N4), REM relative duration	*Entire stimulation night:* no significant results *60-min post-stimulation:* decreased N2, N3 by active vs. sham	NA
Passmann et al. (59)	21 healthy (65.0 ± 1.0 age, 10F) 1 active arm, 1 sham arm, 2/3-week washout	*Electrodes*: 0.5 cm^2^ *Anodes*: 2, DLPFC (F3, F4) *Cathodes:* 2 extra-cerebral (bilateral mastoids) *Intensity*: 0.52 mA/cm^2^ *Type*: alternating 0.75 Hz	*Number*: 1 *Duration*: 30 min (5-min cycles with 1-min free intervals) *Period*: 4 min after N2 onset	*Sleep continuity:* WASO *Sleep architecture:* N1, N2, SWS (N3+N4), REM relative duration	*Entire stimulation night*: decreased N4 by active vs. sham *Stimulation-free intervals*: no significant changes *60-min post-stimulation*: no significant changes	NA
Koo et al. (55)	25 healthy (22.4 ± 2.1 15F) 1 active arm, 1 sham arm, 1-week washout	*Electrodes*: 0.5 cm^2^ *Anodes*: 2, DLPFC (F3, F4) *Cathodes:* 2 extra-cerebral (bilateral mastoids) *Intensity*: NA *Type*: alternating 84 Hz	*Number*: 1 *Duration*: 30 min (5-min cycles with 1-min free intervals) *Period*: during N2	*Sleep continuity:* SE, SOL, TST, TMT, WASO *Sleep architecture:* N1, N2, N3, REM relative duration; REM latency	*Entire stimulation night*: no significant changes	NA
Koo et al. (67)	6 mild cognitive impairment (71.9 ± 9.0 age, 7F) 1 active arm, 1 sham arm, 2-week washout	*Electrodes*: 0.5 cm^2^ *Anodes*: 2, DLPFC (F3, F4) *Cathodes:* 2 extra-cerebral (bilateral mastoids) *Intensity*: 0.52 mA/cm^2^ *Type*: alternating 84 Hz	*Number*: 1 *Duration*: 30 min (5-min cycles with 100-s free intervals) *Period*: during N2	*Sleep continuity:* WASO *Sleep architecture:* N1, N2, N3, N4, REM relative duration	*Entire stimulation night*: no significant changes *Stimulation-free intervals*: Increased N2 by active vs. sham	NA
Saebipour et al. (70)	6 insomnia (34 ± 7 age, 2F) 1 active arm, 1 sham arm, 1-day washout	*Electrodes*: 0.5 cm^2^ *Anodes*: 2, DLPFC (F3, F4) *Cathodes:* 2 extra-cerebral (bilateral mastoids) *Intensity*: NA *Intensity*: alternating 0.75 Hz	*Number*: 1 *Duration*: 30 min (5-min cycles with 1-min free intervals) *Period*: 4 min after N2 onset	*Sleep continuity:* SOL, TST, WASO *Sleep architecture:* N1, N2, N3, REM relative duration	*Entire night after stimulation:* increased probability of transition from N2 to N3, decreased probability of transition from N3 to wakefulness *90-min post-stimulation*: decreased N1 and increased N3 by active vs. sham	
Munz et al. (68)	14 ADHD (12.3 ± 1.4 age) 1 active arm, 1 sham arm, 1-week washout	*Electrodes*: 0.5 cm^2^ *Anodes*: 2, DLPFC (F3, F4) *Cathodes:* 2 extra-cerebral (bilateral mastoids) *Intensity*: 0.52 mA/cm^2^ *Type*: alternating 0.75 Hz	*Number*: 1 *Duration*: 30 min (5-min cycles with 1-min free intervals) *Period*: 4 min after N2 onset	*Sleep continuity:* SE, TST, *Sleep architecture:* N1, N2, SWS (N3+N4), REM relative duration	*Entire night:* no significant changes	NA
Frase et al. (66)	19 insomnia disorder (43.8 ± 15.1 age, 13F) 1 anodal-active arm; 1 cathodal-active arm; 1 sham arm 1-week washout	*Electrodes*: 35 cm^2^ (anode), 100 cm^2^ (cathode) *Anodal-active arm: Anode*: 2 supraorbital areas (Fp1, Fp2)*, Cathode*: 2 parietal (P3, P4) *Cathodal-active arm: Cathode*: 2 supraorbital areas (Fp1, Fp2), *Anode*: 2 parietal (P3, P4) *Intensity*: 2 mA *Current*: direct	*Number*: 2 with 20-min inter-stimulation interval *Duration*: 25 min *Period*: day before night sleep	*Sleep continuity:* Arousal Index, SE, SOL, TST, WASO *Sleep architecture:* N2, SWS (N3), REM relative duration; REM latency, REM cycles,	*Entire stimulation night:* no significant changes	NA

*DLPFC, dorsolateral prefrontal cortex*.

*Sleep architecture: NREM 1–4, Non-Rapid Eye Movement Sleep (four stages of non-rapid eye movement sleep, each progressively going into deeper sleep); REM, Rapid Eye Movement Sleep (unique stage of sleep characterized by random rapid movement of the eyes, low muscle tone throughout the body and propensity of the sleeper to dream vividly)*.

*Sleep architecture (stages) parameters: relative duration, sleep stage proportion during total sleep time (%); latency, time taken for sleep stage onset (minutes); cycles, number of sleep stage occurrence during total sleep time (number count)*.

*Sleep continuity: AI, Arousal Index (number of arousals and awakenings per hour of sleep, in number count); SE, Sleep Efficiency (total time in bed divided by total sleep time in %); SOL, Sleep Onset Latency (time taken for sleep onset in minutes); SWS, Slow Wave Sleep (consists of NREM 3 or NREM 3+4); TST, Total Sleep Time (total time spent asleep in minutes); WASO, Wave After Sleep Onset (time spent awake after sleep onset per night in minutes)*.

#### Results From Studies in Healthy Populations

Fourteen studies investigated the effect of a single brain tES session on sleep patterns in healthy samples (45, 47, 49–52, 55–61, 63, 64). All used a crossover sham-controlled design with days-to-week washout periods between conditions.

Most studies used the Marshall's bifrontal montage (58) with alternating current (49–51, 56–58, 64). Only a minority of studies reported significant results, and these were highly heterogeneous. Over the entire stimulation night, two reports demonstrated an increase in the relative duration of late NREM stages (N3, N4) by active stimulation vs. sham (50, 56). However, a study Passmann et al. found the opposite (59). One study observed a decreased relative duration of stage N1 (50). During stimulation-free intervals, an increased relative duration of stage N2 and wake after sleep onset was found, while later NREM stages were decreased (51, 57). During post-stimulation periods, two studies reported a decrease of N2 and N3 relative duration by active vs. sham (57, 61). A research group that explored the effect of the same montage during REM sleep—in order to trigger conscious awareness in dreams—reported no effect on sleep architecture (63). In parallel, a protocol using bifrontal tES reported a significant decrease of sleep efficiency over the entire stimulation night by alternating stimulation (0.75 Hz) as compared to sham and faster stimulation (5.0 Hz) during REM (45).

Regarding non-bifrontal montages, a single study studied the effect of direct current tES before sleep using fronto-parietal electrode placements (52). These authors reported decreased total sleep time and sleep efficiency when anodes were placed over the frontal cortex and cathodes over the parietal areas, in comparison to the reverse and sham montages. By contrast, REM relative duration was increased by frontal cathodal stimulation in comparison to anodal (52). Finally, a single session of tES during early NREM with the anode overlying the left prefrontal cortex and a contralateral cathode induced no significant changes in sleep architecture parameters of a healthy sample (47).

#### Results From Studies in Clinical Populations

Six studies investigated the effect of a single brain tES session on sleep patterns in clinical samples (65–70). Two crossover sham-controlled studies involved individuals with insomnia disorder. The first demonstrated that a single session of active bifrontal alternating current during early NREM decreases N1 and increases N3 relative duration in the post-stimulation periods as compared to sham (70). The second study showed no significant changes in sleep patterns across the entire night after two fronto-parietal direct current tES sessions during daytime (66). In parallel, a crossover sham-controlled study that gave a single session of bifrontal active alternating current in subjects with ADHD failed to demonstrate significant changes across the entire stimulation night (68). By contrast, a research group that used the same protocol in six elder subjects with mild cognitive impairment found an increase of N2 relative duration during stimulation-free intervals (67). A three-arm study involving participants with fibromyalgia observed a significant sham-controlled effect of five consecutive direct current tES sessions that was specific to the stimulation site, such as anodal stimulation of the left primary motor area increased sleep efficiency and decreased number of arousals, whereas anodal stimulation of the left prefrontal cortex was associated with an increase of sleep latency and REM relative duration, and a decrease in sleep efficiency (69). Finally, a protocol stimulating the affected temporal lobe of patients with temporal lobe epilepsy before a nap demonstrated a significant increase of total sleep time and decrease of sleep onset latency by active vs. sham stimulation (65).

### Subjective Assessments of Sleep

#### Study Designs and Characteristics

Fifteen studies investigated the impact of tES on subjective aspects of sleep in healthy (45, 52, 71–73) and clinical samples (41–44, 46, 48, 66, 74–81). Most used parallel arms (45, 52, 66, 72) or crossover-controlled designs (41–43, 46, 71, 73, 74, 78, 80, 81). The others were uncontrolled series or case reports. Studies details are described in Table 3.

**Table 3:** Impact of transcranial electrical stimulation on subjective aspects of sleep.

**References**	**Design**	**tES montage**	**tES sessions**	**Outcomes**	**Significant findings**	**Adverse effects**
**Placebo-controlled crossover**						
Robinson et al. (72)	21 healthy (20.1, 7F) 1 active anodal arm; 1 sham arm 1-week washout	*Electrodes*: 4 cm^2^ *Anode*: F10 (wake), F3 or F4 (N2/N3 sleep) *Cathode*: extra-cerebral *Intensity*: 2 mA *Current*: alternating	*Number*: 2 (1 wake, 1 sleep) *Duration*: 30 min *Period*: 1 day	KSD	KSD sleep efficiency and sleep quality improvement by active vs. sham at day 1	NA
Johnson and Durrant (45)	15 healthy (20.7 ± 0.3 age, 10F) Active 5-Hz arm Active 0.75-Hz arm Sham arm	*Electrodes*: 2.5 cm^2^ (cathode), 24 cm^2^ (anode) *Cathode*: right DLPFC F4 *Anode*: extra-cranial *Intensity*: 0.4 mA *Current:* alternating (5 Hz or 0.75 Hz)	*Number*: 2 with 20-min inter-stimulation interval *Duration*: 25 min *Period*: during REM sleep	SSS	No significant changes at day 1	1 in the active group (first-degree burn)
Frase et al. (52, 66)	19 healthy (53.7 ± 6.9 age, 13F) 1 active anodal arm; 1 active cathodal arm; 1 sham arm 1-week washout	*Electrodes*: 35 cm^2^ (anode), 100 cm^2^ (cathode) *Active anodal arm:* *Anode*: 2 supraorbital areas (Fp1, Fp2) *Cathode*: 2 parietal (P3, P4) *Active cathodal arm:* *Cathode*: 2 supraorbital areas (Fp1, Fp2) *Anode*: 2 parietal (P3, P4) *Intensity*: 2 mA *Current*: direct	*Number*: 2 with 20-min inter-stimulation interval *Duration*: 20 min *Period*: day before sleep	Subjective sleep parameters/tiredness (VIS-M) and alertness (TAP).	No significant changes at day 3	NA
	19 insomnia disorder (43.8 ± 15.1 age, 13F) 1 active anodal arm; 1 active cathodal arm; 1 sham arm 1-week washout					
**Placebo-controlled parallel-arms**						
Wang et al. (81)	*Active arm:* 31 insomnia disorder (52.5 ± 10.7 age, 24F) *Sham arm:* 31 insomnia disorder (55.3 ± 8.0 age, 23F)	*Electrodes*: anode (42.4 cm^2^), cathode (12.1 cm^2^) *Anode*: Fpz *Cathode*: 2, mastoids *Intensity*: 15 mA *Current:* alternating (77.5 Hz)	*Number*: 20 *Frequency:* daily *Duration*: 40 min *Period*: 4 weeks	PSQI	PSQI improvement by active vs. sham at both week 4 and week 8 Daily disturbance improvement by active vs. sham at week 8	None reported
Charest et al. (71)	*Active arm:* 15 healthy athletes (22.1 ± 1.8 age, 7F) *Sham arm:* 15 healthy athletes (20.1 ± 2.0 age, 8F)	*Electrodes*: 35 cm^2^ *Anode*: (FPz) *Cathode*: (Pz) *Intensity*: 2 mA *Current*: direct	*Number*: 2 *Frequency:* daily *Duration*: 20 min *Period*: 2 days	PSQI ESS ISI ASSQ	PSQI, ISI, and ASSQ improvement by active vs. sham at week 2	NA
Sheng et al. (73)	*Active arm:* 16 healthy (67.6 ± 4.7 age, 9F) *Waiting-list*: 15 healthy (65.8 ± 5.2 age, 10F)	*Electrodes*: High definition *Anode*: left DLPFC (F3) *Cathode*: 4 at 7-cm radius *Intensity*: 1.5 mA *Current*: direct	*Number*: 10 *Frequency:* daily *Duration*: 25 min *Period*: 2 weeks	PSQI	PSQI sleep duration and PSQI sleep efficiency improvement by active vs. sham at week 2	NA
Cody et al. (43)	*Active arm:* 17 HIV (56.0 ± 3.2 age, 6F) *Sham arm*: 16 HIV (55.6 ± 5.4 age, 5F)	*Electrodes*: 4 cm^2^ *Anode*: 1 inferior frontal cortex (F10) *Cathode*: 1 extra-cranial *Intensity*: 2 mA *Current*: direct	*Number*: 15 *Frequency:* every 2–3 days *Duration*: 20 min *Period*: 5 weeks	PSQI	No significant changes at week 5	1 in the active group (first-degree burn)
Acler et al. (41)	*Active arm:* 16 post-polio syndrome *Sham arm*: 16 post-polio syndrome	*Electrodes*: 35 cm^2^ *Anodes*: 1 right premotor cortex (C4), 1 left premotor cortex (C3) *Cathode*: 1 extra-cranial *Intensity*: 1.5 mA *Current*: direct	*Number*: 15 *Frequency:* daily *Duration*: 15 min *Period*: 3 weeks	PSQI	PSQI total improvement by active vs. sham at week 3	1 in the active group (dizziness)
Forogh et al. (74)	*Active arm:* 12 Parkinson disease (61.3 age, 7F) *Sham arm*: 11 Parkinson disease (64.8 age, 7F) 14	*Electrodes*: 35 cm^2^ *Anode*: 1 left DLPFC (F3) *Cathode*: 1 right DLPFC (F4) *Intensity*: 4 mA *Current*: direct	*Number*: 8 *Frequency:* every 1–2 days *Duration*: NA *Period*: 2-weeks	ESS	No significant changes at week 5 and month 3	NA
Harvey et al. (78)	*Active arm:* 6 insomnia disorder (71.0 ± 7.0 age, 11F) *Sham arm*: 8 insomnia disorder (71.0 ± 8.0 age, 6F)	*Electrodes*: 35-cm^2^ *Anode*: primary motor area contralateral to the most painful site (C3/C4) *Cathode*: supraorbital area contralateral to the anode *Intensity*: 2 mA *Current*: direct	*Number*: 5 *Frequency:* daily *Duration*: 20 min *Period*: 1 week	PSQI	No significant changes at week 1 and week 2	None reported
Koo et al. (46)	*Active cathodal arm:* 10 restless legs syndrome (47.3 ± 11.0 age) *Active anodal arm:* 10 restless legs syndrome (44.1 ± 13.4 age) *Sham arm*: 11 restless legs syndrome (46.0 ± 10.1 age)	*Electrodes*: 25 cm^2^ *Active anodal arm: anode*: primary motor area (Cz), *cathode*: extra-cerebral *Active cathodal arm: anode*: extra-cerebral, *cathode*: primary motor area (Cz) *Intensity*: 2 mA *Current*: direct	*Number*: 5 *Frequency:* daily *Duration*: 20 min *Period*: 1 week	PSQI	No significant changes at week 1 and week 2	13 transient but no significant differences between groups
Shill et al. (80)	*Active arm:* 12 Parkinson disease *Sham arm*: 11 Parkinson disease	*Electrodes*: NA *Anodes*: 2 prefrontal areas *Cathodes*: 2 mastoids *Intensity*: 1.5 mA *Current*: direct	*Number*: 10 *Frequency:* daily *Duration*: 45 min *Period*: 2 weeks	ESS	No significant changes at weeks 2, 6, 10 and 14	None
Brunoni et al. (42)	*Sham-tDCS/placebo-pill arm:* 30 depression (46.4 ± 14.0 age, 20F) *Sham-tDCS/sertraline arm:* 30 depression (41.0 ± 12.0 age, 17F *Active-tDCS/placebo-pill arm:* 30 depression (41.0 ± 12.0 age, 21F) *Sertraline-pill arm:* 30 depression (41.0 ± 13.0 age, 24F)	*Electrodes*: anode 42 cm^2^, cathode 12 cm^2^ *Anode*: left DLPFC (F3) *Cathode*: right DLPFC (F4) *Intensity*: 2 mA *Current*: direct	*Number*: 10 *Frequency:* daily + 2 fortnight *Duration*: 30 min *Period*: 6 weeks	MADRS (Sleep item)	No significant changes at week 2	Skin redness rates were higher in the active vs. sham group (25% vs. 8%, *p* = 0.03),
**Uncontrolled**						
Dobbs et al. (44)	12 Parkinson disease (66.9 ± 5.4 age, 4F) Add-on cognitive training	*Electrodes*: 25 cm^2^ *Anode*: left DLPFC (F3) *Cathode*: right DLPFC (F4) *Intensity*: 2 mA *Current*: direct	*Number*: 10 *Frequency:* daily *Duration*: 20 min *Period*: 2 weeks	PROMIS sleep	No significant changes at week 2	Around 50% had mild side effects that resolved after sessions
Hadoush et al. (77)	21 Parkinson disease (62.5 age, 6F)	*Electrodes*: 25 cm^2^ *Anodes*: 1 right primary motor area + DLPFC (FC1) + 1 left (FC2) *Cathode*: 1 right orbitofrontal area (Fp2) + 1 left (Fp1) *Intensity*: 1 mA *Current*: direct	*Number*: 10 *Frequency:* daily *Duration*: 20 min *Period*: 2 weeks	PSQI	PSQI-total and PSQI-sleep latency improvement at week 2	None reported
Minichino et al., (79)	25 bipolar disorder in euthymic state (41.9 ± 12.6 age, 17F)	*Electrodes*: 25 cm^2^ *Anode*: left DLPFC (F3) *Cathode*: right cerebellar cortex *Intensity*: 2 mA *Current*: direct	*Number*: 15 *Frequency:* daily *Duration*: 20 min *Period*: 3 weeks	PSQI	PSQI-total and items (-sleep latency, -sleep quality, -sleep duration, -sleep disturbance, -daytime dysfunction) improvement at week 2	NA
Galbiati et al. (76)	8 idiopathic hypersomnia (35.0 ± 15.5 age, 5F)	*Electrodes*: 25 cm^2^ *Anode*: left DLPFC (F3) *Cathode*: right supraorbital area (Fp2) *Intensity*: 2 mA	*Number*: 12 *Frequency:* three per week *Duration*: 20 min *Period*: 4 weeks	ESS	ESS improvement at week 4 and week 6. No improvement at week 8	NA
**Case reports**						
Frase et al. (75)	1 organic hypersomnia following reanimation (52.0 age, male)	*Electrodes*: 35 cm^2^ (anodes), 100 cm^2^ (cathodes), *Anodes*: 2 Fp1-Fp2 *Cathodes*: 2 P3-P4 *Intensity*: 2 mA *Current*: direct	*Number*: 6 alternated (3 active, 3 sham) *Frequency:* daily *Duration*: 13 min *Period*: 1 week	PVT psychomotor vigilance task	Pre-to-post improvement in response speed by active in comparison to sham at week 1	None
			*Number*: 6 (2 blocks of three active with 4 weeks interval) *Frequency:* daily *Duration*: 1 min *Period*: 4 weeks	VAS subjective vigilance	Significant increase in subjective vigilance and reduction of daytime sleep at week 4	
Sanchez-Kuhn et al. (48)	1 chronic after-stroke dysphagia (64.0 age, male)	*Electrodes*: 35 cm^2^ *Anode*: left M1 (TP-T7) *Cathode*: extra-cerebral *Intensity*: 1 mA *Current*: direct	*Number*: 16 *Frequency:* daily (4 days/w) *Duration*: 20 min *Period*: 4 weeks	SWAL-QoL sleep item	Enhancement in sleep at week 4	Mild itching sensations during the stimulation

*ASSQ, Athlete Sleep Screening Questionnaire; DLPFC, dorsolateral prefrontal cortex; ESS, Epworth Sleepiness Scale; GSAQ, Global Sleep Assessment Questionnaire; ISI, Insomnia Severity Infex; KSD, Karolinska Drowsiness Scale; MADRS, Montgomery–Åsberg Depression Rating Scale; NA, not available; PSQI, Pittsburgh Sleep Quality Index; PVT, Psychomotor Vigilance Task; SE, Sleep Efficiency; SOL, Sleep Onset Latency; SSS, Stanford Sleepiness Scale; SWAL-QoL, Swallowing Quality of Life questionnaire; TAP, Testbatterie zur Aufmerksamkeitsleistung; TST, Total Sleep Time; VAS, Visual Analog Scale*.

#### Results From Studies in Healthy Population

Five studies investigated the impact of a single tES session during wake periods on the subjective sleep parameters in healthy groups. One that used high-definition direct current tES targeting the left prefrontal cortex in elders observed an increase in subjective sleep duration and sleep efficiency in the active group vs. a waiting list group (73). Similarly, a crossover study that tested slow alternating tES during sleep found higher sleep quality and efficiency after active vs. sham stimulation (72). A study that specifically investigated frontal stimulation in athletes observed a global improvement in subjective sleep after two consecutive active stimulation sessions in comparison to a group that received sham stimulations (71). Finally, no significant changes were observed in sleepiness scales neither after two frontal tES sessions during daytime (52) nor during REM sleep (45).

#### Results From Studies in Clinical Populations

All studies used direct current tES (tDCS). Parkinson's disease was the most represented neuropsychiatric disorder. A crossover placebo-controlled trial investigated the effect of 10 consecutive days of tDCS treatment on non-motor symptoms (80). No significant differences between groups were seen in the Epworth Sleepiness Scale (ESS) at week 2 and week 14 following treatment. Similarly, a sham-controlled study that gave eight bifrontal tES sessions reported no significant changes in the Epworth Sleepiness Scale both after the procedure and at long term (74). An uncontrolled study reported an absence of significant effects on the PROMIS^TM^ sleep assessment after 10 sessions of bifrontal tDCS (transcranial direct current stimulation) (44). By contrast, another study observed a significant improvement of the Pittsburgh Sleep Quality Index (PSQI) sleep latency subscore and total score after 10 sessions of a tDCS montage involving two anodes placed between the primary motor area and the prefrontal cortex, and two cathodes placed over the supraorbital areas (77).

In parallel, a single placebo-controlled trial in individuals with post-polio syndrome observed a significant PSQI improvement after 15 consecutive sessions of tDCS with two anodes targeting primary motor areas (41). Besides, studies exploring the impact of tDCS targeting the inferior frontal cortex in HIV patients (43) and targeting the motor areas in patients with restless legs syndrome (46) and chronic pain associated with insomnia (78) yielded negative results.

Regarding mood disorders, a non-controlled tDCS protocol placing the anode over the prefrontal cortex and the cathode over the cerebellum significantly improved sleep quality with 46% improvement of the PSQI in a sample of euthymic patients with bipolar disorder (79). In contrast, no effect of bifrontal tDCS was observed on the sleep item of the Montgomery–Asberg Depression Rating Scale (MADRS) in a randomized controlled trial involving participants with depression (42).

No significant changes were reported for subjective parameters including tiredness and alertness in a crossover study involving two bifrontal tES sessions in patients with insomnia disorder (66). By contrast, a recent randomized placebo-controlled trial demonstrated that 20 daily consecutive sessions of frontal tES with alternating current significantly improved PSQI both after the procedure and at 2 months follow-up (81).

A case series of idiopathic hypersomnia showed that 4 weeks of bifrontal tDCS can reduce excessive daytime sleepiness, as assessed by the ESS (76). Finally, case reports observed daytime vigilance and sleep quality improvement using specific rating scales in organic hypersomnia (75) and after-stroke condition (48), respectively.

## Discussion

Through this systematic literature review, we observe that tES can modify endogenous brain oscillations during sleep and that subjective assessments show clear improvements after tES, while relationships with concomitant changes in sleep architecture warrant validations (see Figure 2 and Supplementary Table 2 for a summary of main results). Tolerability profile of tES appears good with few non-severe side effects reported across studies.

### tES During Sleep Can Be Effective at Modulating Endogenous Oscillations

The main neurophysiological finding of our systematic review is that anodal tES of the frontal areas with a slow alternating or direct current during NREM sleep can immediately enhance the power spectral density (PSD) of endogenous slow oscillations at the stimulated areas (50, 56, 58, 59, 64). Slow oscillatory activity originates in the centro-frontal neocortex and coordinates widespread firing synchrony across other brain regions, including adjacent cortices and subcortical structures such as the thalamus and brainstem nuclei (82–84). This crosstalk is essential for the generation of oscillatory cycles that orchestrate brain activity during sleep (85). Regarding our results, although tES has primarily cortical direct effects, it is likely that external stimulation of the neocortex spreads to the entire cortico-subcortical network through top-down mechanisms to facilitate slow oscillatory activity. This rationale is supported by preclinical results demonstrating that the modulatory effects of electrical stimulation of centro-frontal cortices extend to subcortical arousal networks (86, 87). Moreover, it has been reported that stimulating the prefrontal region can reach deeper structures and lead to subcortical dopamine release in the ventral striatum (88). In parallel, slow alternating stimulation during NREM sleep has been shown to facilitate corticocortical network activity, which can explain the observed enhancement of sleep-dependent restorative processes of tES with respect to declarative memory in healthy participants (45, 47, 50), as well as behavioral inhibition and executive functions in patients with ADHD (68). For more details about the effect of tES during sleep on memory processes, we refer the interested reader to this exhaustive review (37). At the cellular level, it is likely that enhancement of slow oscillatory activity is related to tES-induced anodal polarization, which corroborates previous demonstrations that endogenous negative potentials arising during late stages of NREM sleep and facilitating specific shifts in extracellular ionic concentration play a supportive role in the generation of slow oscillations (84, 89, 90).

In parallel, it was shown that slow oscillation PSD immediately following tES is negatively correlated with measures of slow oscillation spectral power and coherence immediately preceding stimulation, suggesting that tES may be more effective when applied during less synchronized and more “quiet” periods of brain activity (50). This put forward the importance of the baseline cortical activation state on the impact of tES (91). As observed during magnetic stimulation of the brain (92), these findings imply that tES effect interacts with the ongoing activity of the sleeping brain at the time of stimulation.

In addition to modulating corresponding spectral ranges, it was shown that slow oscillatory frontal tES induces collateral modulation and enhancement of spindle waves (47, 49, 56, 59). These observations support the conclusion that slow alternating tES enhances physiologically normal conditions in which slow alternating activity drives the generation of spindle, which implies that spindle activity is maximal during late stages of NREM sleep (i.e., transition to slow wave sleep) (93–95). These neurophysiological changes were also observed during post-stimulation intervals (46, 57, 59, 60), suggesting short-term plasticity effect induced with regard to putative synaptic mechanism consequences described above. Spike timing-dependent plasticity has been proposed for the observed after-effects of tES. According to this form of brain plasticity, when the frequency of an externally induced driving force (i.e., tES) is matched with a neural circuit resonance frequency, spike timing-dependent plasticity can strengthen synapses in this circuit (96). However, changes were not replicated when taking into consideration the entire stimulation night (51), which could be attributed to the decreasing levels of slow oscillations typically observed across a sleep period (97). Regarding lower-frequency ranges, significant delta modulations induced by frontal slow alternating or direct tES during NREM sleep suggest potential cross-frequency coupling mechanisms (51, 53, 58).

### tES Is Mainly Ineffective on Sleep Patterns

Only a minority of studies reported significant changes in sleep architecture, namely, increased sleep stage NREM2 and decreased further slow wave sleep stages relative duration (51, 57, 67), indicating a potential sleep-stabilizing effect of slow oscillatory tES (single session) during NREM sleep. In parallel, an absence of tES beneficial effects on polysomnographic measures of sleep was repeatedly observed across studies conducted in both clinical and healthy samples. It is likely that potential improvement of sleep continuity has been missed due to a ceiling effect in good sleepers. A few studies observed tES-induced sleep continuity disruption, which may be related to potential increase of external disturbance or unwanted modulation of vigilance control circuits (45, 57, 66). Thus, when performing tES especially in the clinical population, stimulation sessions upon awakening should be recommended in order to avoid adverse effects on sleep continuity. Interestingly, a study observed a relative stronger decrease of sleep continuity in healthy controls following tES, but not in patients with insomnia disorder. It is proposed that this differential effect of tES is related to persistent hyperarousal in this clinical population, preventing the arousal-inducing effect of anodal active tES in healthy controls (66).

### tES Can Significantly Improve Subjective Aspects of Sleep

A significant number of studies observed significant improvements of subjective sleep after tES in both healthy (71–73) and a large range of neuropsychiatric diseases (41, 48, 75–77, 79, 81). The positive results were observed across various rating instruments, suggesting large impact of tES on subjective components of sleep. Notably, all studies showing significant improvements used tES montages that stimulated frontal and/or motor areas. This is not surprising since neuroimaging methods identified that cortical topography of slow waves during sleep are primarily associated with activity in a core set of cortical areas that are mainly located in the prefrontal cortex and motor regions (98, 99). Although conventional tES current may spread to other regions than the targeted area (100), studies showing a subjective improvement of sleep after tES used comprehensive stimulation protocols assuring activation of the major cortical areas involved in sleep regulation which, in turn, reflected on subjective sleep measures. It is reported that complementary biological mechanisms of subjective sleep improvement by tES can involve stimulation of the reticular formation (41), which plays a central role in states of consciousness like sleep. In addition, sleep promotion induced by decoupling functional connectivity between wakeful-active default mode network and subcortical structures (73) and activation of non-motor function of the cerebellum such as sleep regulation (101) may underlie subjective sleep improvement by tES.

These results hold promises for the use of tES to target sleep symptoms in various clinical populations with neuropsychiatric disorders. Furthermore, significant correlations were observed between subjective sleep parameters and non-sleep parameters, such as depressive symptoms (77) and attention performance (76). Further investigations are well-warranted to identify if other clinical dimensions are improved through improvement of sleep.

### Negative Results and Limitations

The main limitation of comparisons across reviewed studies relies on the high heterogeneity of results and the important rate of negative results. A main shortcoming that may explain this limitation is the very high heterogeneity of stimulation protocol parameters that could influence the effect on sleep and sleep measures, such as the device type, duration of stimulation on/offsets, electrode impedance, and electrode placement across studies. Slight methodological variations regarding the stimulation signal (direct vs. alternating) and the applied current density may also impact observations. For instance, current ramping at the beginning and at the end of each stimulation interval is likely to influence short-lasting stimulation-dependent entrainments of the specific oscillatory activity (51, 58). In addition, heterogeneity of samples sizes, sleep variations according to age and gender (102, 103), and the considerable interindividual and intraindividual differences commonly observed in sleep recordings may have accounted for the conflicting results. Another potential explanation for inconsistent effects of tES on endogenous oscillations and sleep patterns, which are derived from physiological recordings, is the difficulty in collecting high-quality stable long-term measurements during sleep due to a number of issues such as movement artifacts (rapid eye movements, tossing, turning, etc.) and slippage in the placement of electrodes through the night. As pointed out in a few recent studies (104, 105), the effects of tES on endogenous oscillations can systematically vary through the course of the night due to refractory effects—so future studies need to track stimulation-induced biomarkers for the outcomes of interest during sleep. Finally, no quantitative meta-analyses were applicable due to the high heterogeneity of studies included in this systematic review.

Regarding changes in sleep oscillations, a potential “endogenous” explanation is that the stimulation was likely given in different sleep stages across studies, especially since not all studies controlled stimulation-free intervals of neurophysiological measurement for ongoing sleep stage. Given the interindividual variability of sleep architecture and fragmentation and the fact that oscillatory stimulation effects are strongly dependent on ongoing brain state and network activity, this may have accounted for heterogeneity (106).

Some discrepancies were observed between healthy and clinical samples. It might be that the latter may have different responses to tES with different response/activation threshold, as for instance in insomnia vs. healthy subjects, who met hyperarousal symptoms (66). It is also possible that subjective sleep alterations are secondary to other symptoms such as pain (78), whose neurobiological bases are not directly targeted by tES in sleep-oriented studies. In addition, the strong placebo response observed in neuropsychiatric disorders might have hampered the observation of sleep improvement under active stimulation (80).

Finally, it should be noted that the vast majority of studies included in this systematic review involve frontal stimulation, which prevents us from concluding on a general effect of tES on sleep. A review focusing only on studies stimulating frontal areas could have indeed led to a more homogeneous picture. Nevertheless, we a priori defined the aim of our study to conduct an exhaustive systematic review of tES on sleep with the objective to account for all the existing literature and all possible stimulation areas.

## Conclusion

tES-based approaches have a significant impact on oscillatory neurophysiological parameters of sleep. Furthermore, studies suggest their enhancement as physiological restorative processes that could serve as a potential therapeutic target in neuropsychiatric disorders. While the conflicting effects of tES on sleep patterns shed some doubt on its potential utility to improve sleep continuity, the significance of subjective aspects of sleep in various populations invites further development of non-invasive stimulation treatments for sleep conditions that are among the most prevalent health problems worldwide. Given the important heterogeneity of stimulation protocols and samples, future studies should examine the impact of these variables on the effect of tES on sleep measures. Furthermore, several major questions should be investigated to define optimal application of tES for sleep improvement, in terms of stimulation parameters (e.g., current type, duration, sessions), stimulation location, and type of brain state (e.g., wake/sleep, sleep stage) during stimulation.

## Data Availability Statement

The original contributions presented in the study are included in the article/Supplementary Material, further inquiries can be directed to the corresponding author/s.

## Author Contributions

CD designed the study, performed the literature review, and wrote the first draft of the manuscript. PG supervised the study. JB, J-AM-F, JM, ML, and MP provided critical inputs to the manuscript. All authors reviewed and approved the manuscript in its final form.

## Conflict of Interest

The authors declare that the research was conducted in the absence of any commercial or financial relationships that could be construed as a potential conflict of interest.

## Abbreviations

ADHD: Attention Deficit/Hyperactivity Disorder
EEG: electroencephalography
ESS: Epworth Sleepiness Scale
MeSH: Medical Subject Heading
MADRS: Montgomery-Asberg Depression Rating Scale
NREM: Non-Rapid Eye Movement (sleep)
PRISMA: Preferred Reporting Items for Systematic Reviews and Meta-analysis
PROSPERO: International prospective register of systematic reviews
PSQI: The Pittsburgh Sleep Quality Index
REM: Rapid-Eye Movement (sleep)
QualSyst: Standard Quality Assessment
SWS: Slow Wave Sleep
tACS: transcranial Alternating Current Stimulation
tDCS: transcranial Direct Current Stimulation
tES: transcranial Electrical Stimulation
tRNS: transcranial Random Noise Stimulation.
