# Supplementary material for: The Effects of Transcranial Electrical Stimulation of the Brain on Sleep: A Systematic Review
Source: Front Psychiatry. 2021 Jun 7;12:646569. doi: 10.3389/fpsyt.2021.646569 (PMC8215269; doi:10.3389/fpsyt.2021.646569)
Supplement: Supplementary file 2 [file Data_Sheet_2.PDF]

| Significant effects of frontal stimulation on sleep | Direct current | Alternating current |
|---|---|---|
| | | |
| *Sleep oscillations* | | |
| NREM stimulation | ↑ **So** (Marshall, 2004) | ↑ **So** (Cellini, 2019; Marshall, 2006; Munz, 2015; Passmann, 2016; Saebipour, 2015; Westerberg, 2015) |
| | ↑ **β** (Frase, 2016) | ↑ **Sp** (Ladenbauer, 2017; Marshall, 2006; Passmann, 2016) |
| Post-stimulation | ↑ γ (Frase, 2016) | ↓ **Δ θ** (Eggert, 2013; Garside, 2015) |
| | | ↑ **So, Sa, Sp** (Koo, 2015; Marshall, 2011; Passmann, 2016; Reato, 2013) |
| | | |
| *Sleep patterns* | | |
| NREM stimulation | ↓ **SE** (Roizenblatt, 2007) | ↑ **N2 N3** (Eggert, 2013; Ladenbauer, 2017; Marshall, 2011)<br>↓ **N1** (Cellini, 2019) |
| Post-stimulation | ↑ **REM** (Roizenblatt, 2007)<br>↑ **SOL** (Roizenblatt, 2007) | ↓ **N2 N3** (Marshall, 2011; Sahlem, 2015) |
| Entire stimulation night | ↓ **SE** (Frase, 2016)<br>↓ **TST,** ↑ **WASO** (Frase, 2016) | ↑ **N2 N3 SWS** (Cellini, 2019; Marshall, 2006)<br>↓ **N1** (Cellini, 2019)<br>↓ **N4** (Passman, 2014)<br>↓ **SE** (Johnson and Durrant, 2018) |
| | | |
| *Subjective effects* | | |
| Subjective sleep improvement | ↑ **subjective parameters** (Sheng, 2018). | ↑ **subjective parameters** (Charest, 2019; Robinson, 2018) |

**Supplementary Material S2**: Highlights of major significant effects of frontal tES on sleep with corresponding references. Results regarding non-frontal tES, tES during REM and fast alternating tES were scarce, and thus omitted from the Table.

Effect on sleep patterns: NREM = non rapid eye movement sleep; N1-4 = NREM sleep stages relative duration; REM = rapid eye movement sleep; SE = sleep efficiency; SWS = slow wave sleep relative duration; SOL = sleep onset latency; WASO = wake after sleep onset.
Effects on sleep oscillations: β = beta power spectral density PSD (15-25 Hz); Δ = delta PSD (1-4 Hz); γ = gamma PSD (> 25 Hz); θ = theta PSD (4-8 Hz); NREM = non-rapid eyes movement sleep; So = slow oscillations PSD (0.5-1 Hz); Sa = slow activity PSD (0.5-4 Hz); Sp = spindle PSD (10-15 Hz).
